# Annotations

**Published:** 1888-01-14

**Authors:** 


### ANNOTATIONS.

**The Women's Jubilee Offering.**

AFTER much deliberation, the Committee appointed to advise her Majesty as to the ultimate destination of the large surplus of the Women's Jubilee Offering have arrived at a decision. That decision is one which can hardly fail to commend itself at once to all who have practical acquaintance with the needs and distresses of the poor. Whilst the question was still *sub judice*, we hoped a portion of the seventy thousand pounds remaining for distribution might have been put to an excellent use by being devoted to the foundation of a National Pension Fund for trained nurses. But the object which the Committee have finally decided to commend to her Majesty includes a vast region of work which has hitherto lain almost entirely outside the efforts of an active philanthropy, and is in itself so excellent and so large and various in its possibilities that we not only give our most cordial adherence to it, but think it abundantly worthy of the seventy thousand pounds at disposal, and of as much more as a generous and sympathising public may find it in their hearts to give. The money is to be spent in founding an institution for the training and maintenance of a staff of nurses for the gratuitous service of the sick poor at their own homes. The idea is full of a large humanity, and being essentially humane is essentially wise. A kind and gentle woman in a home reduced to confusion and desolation by the helpless break-down of the mother, is simply of priceless value. If, in addition to kindness and gentleness, she possesses knowledge and trained capacity, the best is done for the distressed family which the highest Christian sentiment can desire. It was a truly patriotic and statesman-like instinct which prompted the committee to include Edinburgh and Dublin within the range of their proposed operations. In each of those capital cities, as well as in the metropolis, we may now expect an institution to be founded which shall extend its deeds of blessing over the whole land. We think it emphatically desirable that in Edinburgh and in Dublin, as well as in London, the institution shall bear the honoured name of Queen Victoria. It would be difficult to imagine any permanent memorial of the Queen's jubilee more worthy of the unique occasion.

**The "Times" Centenary.**

LAST year the Jubilee of Queen Victoria was celebrated; this year marks the centenary of the *Times* newspaper. We venture, as representing hospitals and other voluntary charities, to offer our most cordial congratulations to Mr. Walter and all connected with the Leviathan of the Press on the great occasion. The *Times* has now been in existence for upwards of a hundred years, and during the greater part of that time—perhaps during the whole of it—has been a steady and consistent friend to hospitals and other voluntary charities. Out of 70 hospitals, whose names appear in the Almanac we issued last week, only seven general hospitals were in existence when the *Times* was first established, and there were no special hospitals at all, as those institutions are now understood. The seven general hospitals were: St. Bartholomew's, Guy's, St. Thomas's, The London, St. George's, The Middlesex, and Westminster. In addition, there were three lying-in hospitals—the General, the British, and the City of London—and also the Lock. As against this there are now upwards of seventy general and special hospitals, besides a host of other voluntary charities of all kinds. It may readily be imagined how all these institutions, struggling and striving for existence, besiege every editor's room in London for notice and help. The claims made are so numerous and urgent, and some of the claimants have so little to plead on their own behalf, that we cannot be surprised if certain editors decline absolutely to deal with them in any way. The *Times*, however—and we have had personal experience of three editors—has always given the necessary labour to the sifting of the wheat from the chaff, and has done its utmost, even at periods of the greatest political pressure, to promote, encourage, and develope in every possible way those institutions which really strive to meet the public needs. The service thus rendered has been of the greatest possible value, and many a deserving but struggling charity has been helped over desperate emergencies by such timely aid. We are sure that all hospital officials of any lengthened experience will endorse this simple statement of facts, and will unite with us in the sincerest congratulations to our powerful and world-famous contemporary.

**The Origin of Genius.**

MODERN biography begins as ancient fiction used to do, not with the hero's self, but with his ancestors his "forebears," as the Scotch, the most genealogical of nations, term them. If the biographer is merciful, he starts us with a comparatively recent progenitor, the great grandfather, or the maternal aunt; but the more thoroughgoing narrator takes us back to the ancestor who "drew a good bow at Hastings," and the legendary collateral relative on the distaff side who "died of love and green apples." Of course, those ancient and sometimes mythical kindred are brought in to illuminate some unexpected point in the hero's character—the combination of warlike spirit with feminine sensitiveness, and a lurking tendency to vegetarianism. The science of heredity is ever with us, and we cannot observe the frown or smile of a great man without asking curiously, "How came it there?" There is no doubt that this plan is useful for the biographer. There are some men of whom Lives are written, of whose ancestors more can be said than of themselves. The fighting forefather is decidedly picturesque; but when you have evolved your Frankenstein-hero from the relics of his dead and gone relations, your science of heredity fails you. It may show you why he is—what he is; but it cannot explain why his brothers and sisters, blessed with the same ancestors and brought up in the same environment, are not like him. They ought to be, by logical deduction—from similar causes similar effects should spring; but they don't. Isaac Evans, the brother of George Eliot, had no very subtle comprehension of the mysteries of the human heart; indeed, seems to have been a rather dull and narrow-minded mortal. Gilbert Burns was an unsuccessful farmer, like his brother Robert, but he was not therefore a poet too. Alfieri was a strangely phenomenal growth to spring from the narrow-minded and conservative aristocracy of eighteenth-century Italy; none felt it more than his own family. In some families, indeed, we see just enough talent in each member to have made a genius, if united in the person of one; in others the combination is effected—there is one great man, the pride, glory, and envy of half-a-dozen nonentities. Nature is sometimes fair all round, and sometimes does as skilful gardeners do, and sacrifices the numerous indifferent blossoms to produce one of completer beauty. After all, genius is a "gift enterprise"—a lottery; it does not come where most expected, and appears in the most unlooked-for places. When it has come we can see, or think we can see, whence it was derived; but we cannot predict its advent from pre-existing facts, nor say why, other things being equal, its appearance is not invariable. It is always the way. When we have got our cut-and-dry system, our infallible solution of the mysteries of the universe, there comes along a mystery that declines to be solved by it. Perhaps we are too fond in this age of thinking that we know the whole order and method of creation. The origin of superior talents, like some other matters of higher import and one or two of slighter value, cannot yet be set down as a geometrical problem which we can explain and finish off with a triumphant *Q. E. D.*

